# Isthmic papillary thyroid microcarcinoma: clinicopathological features and prognostic factors following isthmusectomy based on tumor size stratification

**DOI:** 10.1007/s12672-026-04950-0

**Published:** 2026-04-05

**Authors:** Feng Zhu, CuiWei Li, YiBin Shen, MengXia Li, XueYu Zhou, YiJun Wu

**Affiliations:** The Department of Thyroid Surgery, The First Affiliated Hospital, School of Medicine, Zhejiang University, 79# Qingchun Road, Hangzhou, 310003 China

**Keywords:** Isthmic papillary thyroid microcarcinoma, Tumor size, Isthmusectomy, Clinicopathological features, Recurrence

## Abstract

**Background:**

This study aimed to compare clinicopathological features and recurrence risk in clinically node-negative (cN0) solitary isthmic papillary thyroid microcarcinoma (PTMC) ≤ 5 mm and > 5 mm in diameter treated with isthmusectomy, and to identify predictors of recurrence.

**Methods:**

A retrospective review was performed of 201 cN0 patients with solitary isthmic PTMC who underwent isthmusectomy between 2018 and 2024. Patients with preoperative high-risk features were excluded. Subgroup analysis compared clinicopathological characteristics between the ≤ 5 mm (*n* = 107) and > 5 mm (*n* = 94) groups. Univariate and multivariate Cox models were used to identify factors associated with recurrence-free survival (RFS).

**Results:**

Significant differences were observed between the two groups in tumor size (*p* < 0.001), central lymph node metastasis (CLNM) (*p* = 0.009), microscopic extrathyroidal extension(mETE) (*p* < 0.001), and BRAF^V600E^ mutation (*p* = 0.008). During follow-up, 9 patients (4.5%) developed recurrence. Recurrence was significantly associated with tumor size > 5 mm (*p* = 0.028), CLNM (*p* = 0.002), and a higher number of metastatic central lymph node (CLN) (*p* < 0.001). Receiver operating characteristic analysis identified metastatic CLNs > 3 as the optimal cut-off for predicting recurrence. Kaplan–Meier analysis demonstrated worse RFS in patients with tumors > 5 mm (*p* = 0.031). Multivariate analysis confirmed metastatic CLNs > 3 as an independent predictor of recurrence (HR 5.298, 95% CI 1.473–19.060, *p* < 0.001).

**Conclusion:**

This study demonstrated that isthmic PTMC tumors > 5 mm were associated with a higher risk of recurrence. In addition, CLNM, especially a metastatic CLNs > 3, may have prognostic significance and should be considered in surgical decision-making for isthmic PTMC.

## Introduction

Thyroid cancer is the most common endocrine malignancy, with papillary thyroid carcinoma (PTC) accounting for approximately 80–85% of cases [1]. Although PTC most commonly arises in the thyroid lobes, approximately 2.2%–12.3% of cases originate in the isthmus, making isthmus PTC relatively uncommon but not rare [2, 3]. Compared with PTCs located in the lobes, isthmus PTCs are more frequently associated with extrathyroidal extension(ETE), multifocality, and central lymph node metastasis (CLNM) [4–7].

Surgery remains the first-line treatment for thyroid cancer. Traditionally, total thyroidectomy with bilateral prophylactic central compartment neck dissection has been recommended for isthmic PTC because of its more aggressive behavior [7–9]. For low-risk isthmus PTC, isthmusectomy has been suggested as a less invasive option that preserves thyroid function and reduces morbidity [2, 10, 11]. Nevertheless, the clinical significance and optimal management of isthmic PTMC remain controversial. The recently published 2025 American Thyroid Association (ATA) guidelines for adult patients with differentiated thyroid cancer recommend more conservative strategies for selected patients [12]. The average thickness of the thyroid isthmus is 3.4 ± 1.7 mm, with the thickest portion measuring approximately 5 mm [13]. Isthmic PTMC larger than 5 mm tend to exhibit more aggressive features, such as CLNM and invasion of surrounding tissues. Thus, stratifying isthmic PTMCs using a 5 mm threshold may have practical clinical relevance. Our previous study demonstrated that recurrence rates were comparable between patients with isthmic PTMCs ≤ 5 mm and those > 5 mm who underwent total thyroidectomy [5]. However, to our knowledge, the clinicopathologic features and prognostic risk factors following isthmusectomy of isthmic PTMCs ≤ 5 mm and those > 5 mm remain undetermined.

Therefore, the objective of this study was to evaluate the clinicopathologic features and oncologic outcomes in patients with isthmic PTMCs ≤ 5 mm and > 5 mm who underwent isthmusectomy at our institution. Additionally, we aimed to identify risk factors for CLNM and recurrence in order to refine patient selection and optimize surgical decision-making.

## Materials and methods

### Patients

A total of 201 patients with isthmic PTMC (139 women and 62 men) who underwent isthmusectomy with bilateral central neck dissection between February 2018 and February 2024 at the Department of Thyroid Surgery, The First Affiliated Hospital, School of Medicine, Zhejiang University, were retrospectively analyzed. Isthmic PTMC was defined as a tumor with its center located between two imaginary perpendicular lines drawn from the most lateral borders of the trachea. Preoperatively, all patients underwent ultrasound-guided fine-needle aspiration biopsy (FNAB). Clinicopathological characteristics were collected and analyzed. Patients were classified into two subgroups according to tumor size: isthmic PTMC ≤ 5 mm (*n* = 94) and isthmic PTMC > 5 mm (*n* = 107). This study was approved by the Clinical Research Ethics Committee of the First Affiliated Hospital, School of Medicine, Zhejiang University (No. IIT20251235A), and written informed consent was obtained from all participants.

### Inclusion and exclusion criteria

Patients were eligible for inclusion if they had a solitary isthmic PTMC without evidence of central or lateral lymph node metastasis (cN0) or gross extrathyroidal extension (gETE). The exclusion criteria were as follows: (1) concurrent other surgical procedures; (2) previous neck surgery; (3) other pathological types of thyroid cancer; (4) distant metastasis; (5) a family history of thyroid cancer; and (6) a history of neck irradiation. If gETE was identified intraoperatively, the surgical procedure was converted to total thyroidectomy. The extent of isthmusectomy was defined as the removal of thyroid tissue located above the horizontal plane at a depth of 0.5 cm from the anterior surface of the trachea. Bilateral level VI lymph node dissection was also performed. CLNM was defined as the presence of at least one pathologically confirmed metastatic lymph node in the central compartment (level VI). The number of metastatic CLNs and dissected CLNs was determined based on postoperative pathological reports, and all counts referred to the total number of nodes retrieved from bilateral central neck dissection. Microscopic ETE (mETE) was defined as tumor extension identified on microscopic examination, in which tumor cells were observed beyond the anatomical boundary of the thyroid gland, with invasion into the surrounding perithyroidal soft tissue. Each pathological diagnosis is initially issued by one pathologist and subsequently reviewed by another pathologist.

### Follow-up

All patients received postoperative thyroid-stimulating hormone (TSH) suppression therapy. Considering the relatively higher recurrence tendency of isthmic PTC compared with tumors located in the thyroid lobes, all patients were managed using a uniform treatment strategy. The target TSH level was 0.1–0.5 mU/L during the first postoperative year and 0.5–2.0 mU/L thereafter, with dynamic adjustments based on follow-up findings. Postoperative physical examinations were performed every 3 to 6 months. The mean follow-up duration was 30.7 ± 11.9 months (median, 30.0 months). Cervical CT and FNAB were performed during follow-up to evaluate suspected recurrence. Patients with suspected recurrence underwent reoperation, and the diagnosis was confirmed via postoperative pathological examination.

### Statistical analysis

Statistical analyses were performed using SPSS version 22.0. Continuous variables were expressed as the mean ± standard deviation (SD), and categorical variables were compared using the chi-square test or Fisher’s exact test, as appropriate. Differences in continuous variables between groups were assessed using Student’s t-test. Multivariable Cox proportional hazards regression analysis was performed to identify independent prognostic factors for recurrence. The optimal cut-off point was determined using receiver operating characteristic (ROC) curves. Recurrence-free survival (RFS) was estimated using the Kaplan–Meier method, and differences between groups were compared using the log-rank test. Results were reported as hazard ratios (HRs) with 95% confidence intervals (95% CIs). A *p*-value < 0.05 was considered statistically significant.

## Results

### Demographic and tumor characteristics

This study included 201 patients with a mean age of 43.35 ± 11.94 years (range, 21–77 years) at the time of diagnosis. The clinicopathologic features of the patients with isthmic PTMC are summarized in Table 1. Among the patients, the male-to-female ratio was 1:2.24, and 20.4% were aged ≥ 55 years. The mean tumor size was 0.59 ± 0.20 cm, and the proportions of tumors ≤ 5 mm and > 5 mm were 46.8% and 53.2%, respectively. The rates of mETE and CLNM in isthmic PTMCs were 56.7% and 30.3%, respectively. All patients underwent bilateral CLN dissection, with a mean of 6.99 ± 4.50 dissected CLNs and a mean of 0.94 ± 1.93 metastatic CLNs. Among all patients with CLNM, metastatic foci ≥ 2 mm were identified in 17 of 61 patients (27.9%), and the maximal size of metastatic foci was 1.36 ± 1.10 mm. Regarding postoperative complications, no patients experienced hoarseness. In addition, neither transient nor permanent hypocalcemia was observed in this cohort.

**Table 1:** Clinicopathologic characteristics of isthmic papillary thyroid microcarcinoma patients

Characteristic	Total (*n* = 201)
Sex	
Male, n (%)	62 (30.8%)
Female, n (%)	139 (69.2%)
Age, years, mean ± SD (range)	43.35 ± 11.94( 21–77)
<55 year	160 (79.6%)
≥55 year	41 (20.4%)
Tumor size, cm, mean ± SD (range)	0.59 ± 0.20(0.3-1.0)
≤5 mm, n (%)	94 (46.8%)
>5 mm, n (%)	107 (53.2%)
CLNM, n (%)	
Yes	61 (30.3%)
No	140 (69.7%)
mETE, n (%)	
Yes	114 (56.7%)
No	87 (43.3%)
Mean no. dissected CLN	6.99 ± 4.50
Mean no. metastatic CLN	0.94 ± 1.93
Ratio of CLNM	0.13 ± 0.23
Maximal size of metastatic foci (mm), mean ± SD	1.36 ± 1.10
Metastatic foci ≥ 2 mm, n (%)	17/61 (27.9%)
BRAF^V600E^ mutation, n (%)	150 (74.6%)
Hoarseness n (%)	0 (0%)
Hypocalcemia	
Transient n (%)	0 (0%)
Permanent* n (%)	0 (0%)
Recurrence, n (%)	9 (4.5%)
Follow-up time, months, mean(range)	30.7 ± 11.9(12–88)

CLN, central lymph node; mETE, Microscopic Extrathyroidal Extension; CLNM, Central Lymph Node Metastasis

*: Permanent hypocalcemia was defined as persistently decreased serum calcium levels requiring ongoing calcium supplementation and calcitriol therapy for more than 6 months after surgery

### Clinicopathologic characteristics by tumor size

Table 2 compares the clinical characteristics between the ≤ 5 mm and > 5 mm groups. There were no significant differences in the proportion of female patients or in age between the two groups. The mean tumor size was 0.42 ± 0.09 cm in the ≤ 5 mm group and 0.75 ± 0.13 cm in the > 5 mm group. The rates of mETE (39 of 94 [41.5%] vs. 75 of 107 [70.1%]; *p* < 0.001), CLNM (20 of 94 [21.3%] vs. 41 of 107 [38.3%]; *p* = 0.009), and BRAF^V600E^ mutation (62 of 94 [66.0%] vs. 88 of 107 [82.2%]; *p* = 0.008) were significantly lower in the ≤ 5 mm group than in the > 5 mm group. The mean numbers of metastatic CLNs and dissected CLNs in the ≤ 5 mm group were significantly lower than those in the > 5 mm group (0.46 ± 1.43 vs. 1.36 ± 2.20, *p* < 0.001, and 6.29 ± 3.92 vs. 7.61 ± 4.89, *p* = 0.038, respectively). Metastatic foci ≥ 2 mm were observed in 5 of 20 patients (25.0%) in the ≤ 5 mm group and 12 of 41 patients (29.3%) in the > 5 mm group, with no significant difference between the two groups (*p* = 0.568).

**Table 2:** Clinical characteristics of iPTMC patients in ≤ 5 mm and > 5 mm groups

Characteristic	≤ 5 mm group (*n* = 94)	> 5 mm group (*n* = 107)	*p* value
Female, n (%)	65(69.1%)	74(69.2%)	0.999
Age (years), mean ± SD	43.79 ± 11.41	42.97 ± 12.42	0.630
Age < 55 years, n (%)	75(79.8%)	85(79.4%)	0.951
Tumor size (cm), mean ± SD	0.42 ± 0.09	0.75 ± 0.13	**< 0.001**
CLNM, n (%)	20(21.3%)	41(38.3%)	**0.009**
mETE, n(%)	39(41.5%)	75(70.1%)	**< 0.001**
Mean no. dissected CLN	6.29 ± 3.92	7.61 ± 4.89	**0.038**
Mean no. metastatic CLN	0.46 ± 1.43	1.36 ± 2.20	**< 0.001**
Ratio of CLNM	0.07 ± 0.17	0.18 ± 0.27	**< 0.001**
> 3 MNCND	2(2.1%)	20(18.7%)	**< 0.001**
Maximal size of metastatic foci (mm), mean ± SD	1.26 ± 1.28	1.41 ± 0.99	0.622
Metastatic foci **≥** 2 mm, n (%)	5/20(25.0%)	12/41(29.3%)	0.568
BRAF^V600E^ mutation, n(%)	62(66.0%)	88(82.2%)	**0.008**
Recurrence, n (%)	1(1.1%)	8(7.5%)	**0.028**

CLN, central lymph node; mETE, Microscopic Extrathyroidal Extension; CLNM, Central Lymph Node Metastasis

The bolded *p*-values represent statistically significant

### Recurrence and prognostic factor analysis

**Fig. 1:**
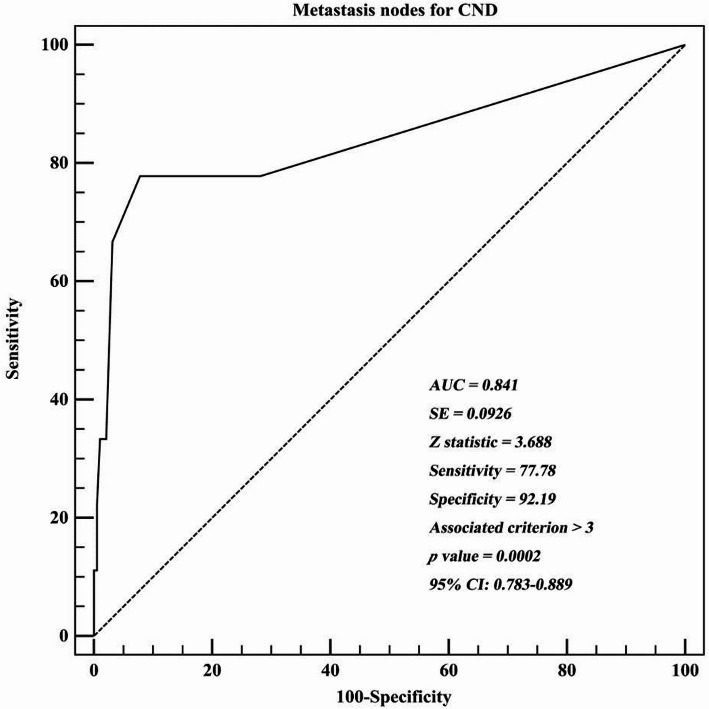
Receiver operating characteristic (ROC) curve analyses of the numbers of lymph nodes metastases for predicting recurrence. CND, Central neck dissection

During the follow-up period, nine (4.5%) patients developed recurrence. Recurrences were observed in the residual thyroid in 2 patients (22.2%), the central compartment in 1 patient (11.1%), both the residual thyroid and central compartment in 1 patient (11.1%), the lateral compartment in 2 patients (22.2%), and both the central and lateral compartments in 3 patients (33.3%). The mean time to recurrence after surgery was 21.6 ± 9.3 months (range, 12 to 40 months). In the ≤ 5 mm and > 5 mm groups, the recurrence rates were 1.1% and 7.5%, respectively. Compared with the 192 patients without recurrence, the 9 patients who experienced recurrence had a higher proportion of tumors > 5 mm (*p* = 0.028), a higher rate of CLNM (*p* = 0.002), and a higher number of metastatic CLNs (*p* < 0.001; Table 3). Comparison between the recurrence and non-recurrence groups demonstrated a significant association between metastatic focus size and recurrence. Metastatic foci ≥ 2 mm were present in 4 of 7 patients (57.1%) in the recurrence group compared with 13 of 54 patients (24.1%) in the non-recurrence group. In addition, the maximal size of metastatic foci was significantly larger in the recurrence group (2.42 ± 1.59 mm vs. 1.24 ± 0.98 mm), with both comparisons reaching statistical significance (*p* = 0.004 and *p* = 0.011, respectively). An ROC curve analysis was performed to assess recurrence prediction based on the number of metastatic CLNs (Fig. 1). The area under the ROC curve (AUC) was 0.841 (*p* = 0.0002), indicating that the number of metastatic CLNs could accurately predict recurrence in isthmic PTMCs. According to Youden’s index, the optimal cutoff value for metastatic CLNs was > 3. The sensitivity and specificity were 77.78% and 92.19%, respectively, with a 95% CI of 0.783–0.889. Multivariable analysis indicated that having metastatic CLNs > 3 was an independent prognostic factor associated with poor RFS (HR = 5.298 [95% CI: 1.473–19.060]; *p* < 0.001) (Table 4).

**Table 3:** Clinicopathologic factors related to tumor recurrence

Characteristic	Recurrence (*n* = 9)	No recurrence (*n* = 192)	*p* value
Female, n(%)	7 (77.8%)	132 (68.8%)	0.567
Age (years), mean ± SD	39.11 ± 8.21	43.55 ± 12.06	0.276
Age < 55 years, n(%)	0 (0%)	41 (21.4%)	0.120
Tumor size (cm), mean ± SD	0.69 ± 0.15	0.59 ± 0.20	0.142
Tumor size≤5 mm, n(%)	1 (11.1%)	93 (48.4%)	**0.028**
CLNM, n (%)	7 (77.8%)	54 (28.1%)	**0.002**
mETE, n (%)	5 (55.6%)	109 (56.8%)	0.943
Mean no. dissected CLN	8.56 ± 4.59	66.92 ± 4.49	0.286
Mean no. metastatic CLN	5.22 ± 3.87	0.73 ± 1.53	**< 0.001**
Ratio of CLNM	0.53 ± 0.35	0.11 ± 0.21	**< 0.001**
> 3 MNCND, n(%)	7 (77.8%)	15 (7.8%)	**< 0.001**
Maximal size of metastatic foci (mm), mean ± SD	2.42 ± 1.59	1.24 ± 0.98	**0.011**
Metastatic foci **≥** 2 mm, n (%)	4/7 (57.1%)	13/54 (24.1%)	**0.004**
BRAF^V600E^ mutation, n(%)	9 (100.0%)	141 (73.4%)	0.073
Site of recurrence, n (%)			
Residual thyroid	2 (22.2%)	0 (0%)	–
Central compartment	1 (11.1%)	0 (0%)	–
Residual thyroid and central compartment	1 (11.1%)	0 (0%)	–
Lateral compartment	2 (22.2%)	0 (0%)	–
Central and lateral compartment	3 (33.3%)	0 (0%)	–

CLN, central lymph node; mETE, Microscopic Extrathyroidal Extension; CLNM, Central Lymph Node Metastasis

The bolded *p*-values represent statistically significant

—: Statistical comparison was not performed because no events were observed in one or more groups

**Table 4:** Univariate and multivariate analysis of variables associated with tumor recurrence

Variables	Univariate		Multivariate	
	HR(95% CI)	*p* value	HR(95% CI)	*p* value
Sex (vs. female)	1.639 (0.340–7.897)	0.538		
Age (vs. < 55y)	0.036 (0.000-53.324)	0.372		
mETE (vs. no extension)	1.015 (0.271–3.803)	0.983		
BRAF^V600E^ mutation (vs. no mutation)	32.997 (0.063-17284.095)	0.274		
Tumor size (vs. ≤5 mm)	7.090 (0.886–56.704)	0.065	1.761 (0.170-18.194)	0.635
Metastatic CLN (vs. ≤ 3)	31.201 (6.475-150.344)	**< 0.001**	5.298 (1.473–19.060)	**< 0.001**

CLN, central lymph node; mETE, Microscopic Extrathyroidal Extension

The bolded *p*-values represent statistically significant

**Fig. 2:**
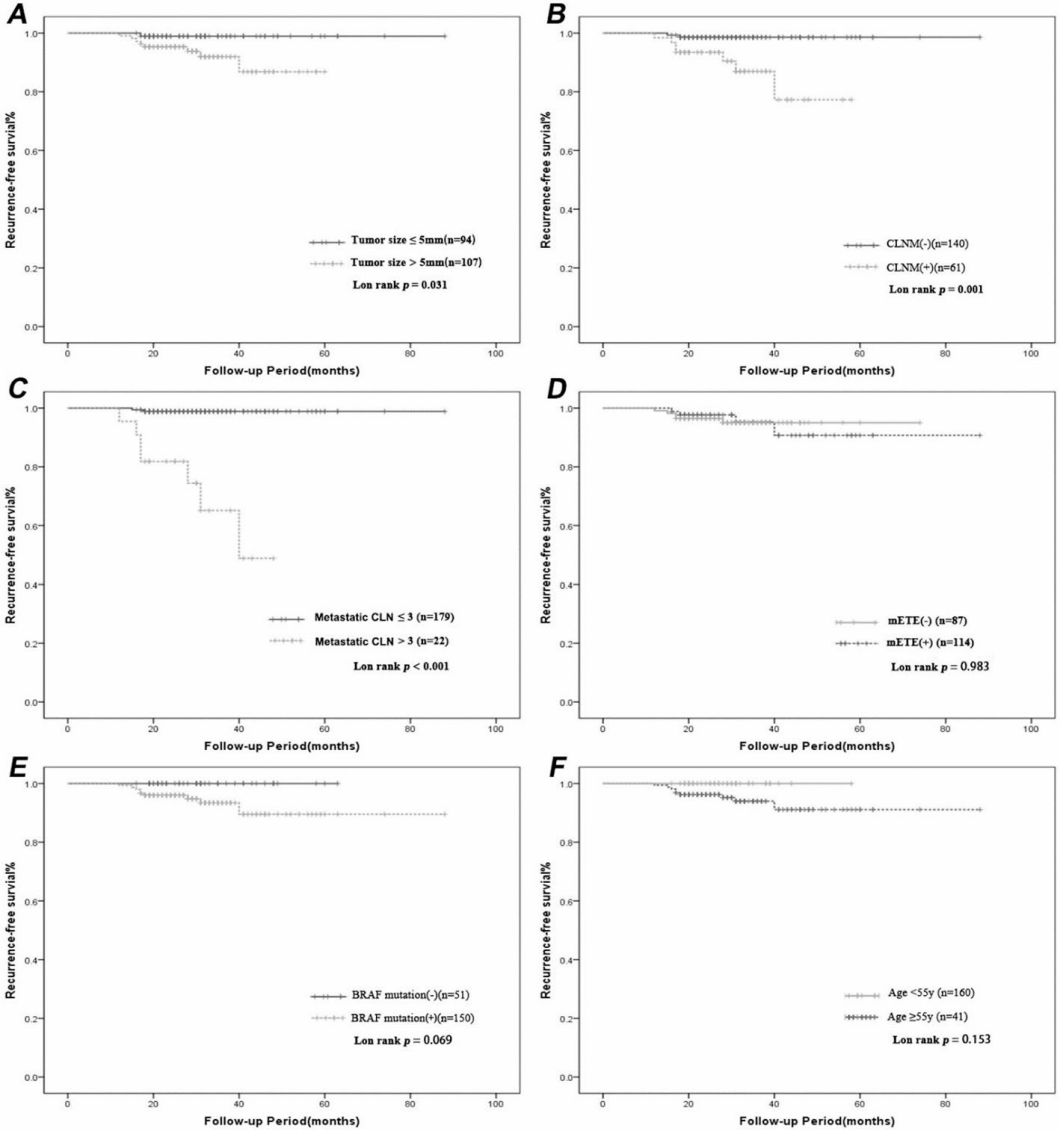
Recurrence-free survival (RFS) according to tumor size (≤ 5 mm and > 5 mm) (**A**); CLNM-negative (CLNM-) and CLNM-positive (CLNM+) (**B**); Metastatic CLN (> 3 and ≤ 3) (**C**); mETE-negative (mETE-) and mETE-positive (mETE+) (**D**); BRAF^V600E^ mutation-negative (BRAF^V600E^ mutation-) and BRAF^V600E^ mutation-positive (BRAF^V600E^ mutation+) (**E**) and age (< 55y and ≥ 55y) (**F**) in isthmic PTMCs. The Kaplan–Meier method for recurrence with the log-rank test was used for statistical comparisons. CLNM, Central Lymph Node Metastasis; CLN, Central Lymph Nodes; mETE, Microscopic Extrathyroidal Extension

Kaplan–Meier survival analysis with the log-rank test was performed to evaluate prognosis (Fig. 2). Compared with the tumor size ≤ 5 mm and CLNM-negative groups, the tumor size > 5 mm and CLNM-positive groups had significantly lower RFS (*p* = 0.031 and *p* = 0.001, respectively; Fig. 2A and B). The RFS was significantly lower for patients with > 3 metastatic CLNs (*p* < 0.001; Fig. 2C). The mETE-negative and mETE-positive groups did not show statistical differences in recurrence (*p* = 0.983; Fig. 2D). There were no significant differences between BRAF-positive and BRAF-negative groups (*p* = 0.069) or between patients aged < 55 and ≥ 55 years (*p* = 0.153) (Fig. 2E and F).

## Discussion

PTC is the most common endocrine malignancy, accounting for approximately 85%–90% of all thyroid cancers [14, 15]. Generally, PTC is considered an indolent tumor with an excellent prognosis. The overall 10-year survival rate for stage I PTC exceeds 95% [16]. The thyroid isthmus is anatomically located between the trachea and the infrahyoid muscles, with a reported average thickness of approximately 3.4 ± 1.7 mm, and generally around 5 mm in healthy adults [13]. Given this limited anteroposterior dimension, relatively larger isthmic tumors may be more likely to contact the anterior or posterior thyroid capsules, potentially increasing the likelihood of microscopic capsular penetration and the subsequent detection of mETE. This anatomical consideration also provided a rationale for using 5 mm as the cut-off value for subgroup analysis in the present study. Isthmic PTC has been reported to account for approximately 2.2%–12.3% of all PTC cases [2, 3]. Nevertheless, isthmic PTCs have consistently been reported to exhibit higher rates of ETE, CLNM, and multifocality than PTCs arising in the lateral lobes. Lee et al. observed that the rates of ETE and CLNM in isthmic PTC were 100% and 71.4%, respectively, while Hahn et al. reported rates of 83.3% and 68.8%, respectively [2, 17]. Goldfarb et al. demonstrated that the incidence of multifocality in isthmic PTC was 67% [18]. Accordingly, total thyroidectomy has traditionally been regarded as the standard surgical approach for isthmic PTC due to its potentially aggressive biological behavior and the objective of achieving complete oncologic control [6, 19].

However, in recent years, treatment paradigms for differentiated thyroid carcinoma have shifted toward more conservative strategies [1]. The 2025 ATA guidelines recommend lobectomy over total thyroidectomy for low-risk tumors measuring up to 4 cm without gETE or other high-risk features [12]. Accordingly, debate has emerged as to whether more conservative surgical approaches, such as isthmusectomy, represent viable alternatives for isthmic PTC [11, 20]. Wu et al. found that wide-field isthmusectomy may be a viable surgical option for selected patients with isthmic PTC [21]. Park et al. reported 43 patients with isthmic PTC and concluded that isthmusectomy alone was an acceptable option for selected low- and intermediate-risk cases [22]. Thyroid isthmusectomy has been proposed as a surgical option for isthmic PTC, aiming to balance oncologic control with preservation of thyroid function. By preserving both thyroid lobes, this approach may reduce procedure-related morbidity and the need for lifelong levothyroxine therapy [23–25]. Despite these potential advantages, patient selection for isthmusectomy should be approached with caution, as high-risk histopathologic features may be identified postoperatively. Previous studies have shown that a subset of patients considered suitable for conservative surgery may harbor occult aggressive features, including high-risk variants, lymphovascular invasion, or mETE. In such cases, these findings may prompt additional treatment, including completion thyroidectomy [26]. In the present study, we aimed to reduce the risk of pathological upstaging by applying strict inclusion criteria, focusing on patients with solitary isthmic PTMC without preoperative evidence of nodal metastasis or gross extrathyroidal extension.

Although PTMC is generally indolent, larger tumors in the thyroid isthmus are more likely to exhibit invasive features and nodal metastasis due to the thin isthmic anatomy. Therefore, isthmusectomy may be an appropriate surgical option for selected patients with isthmic PTMC, although the optimal criteria for patient selection remain to be clearly defined. Thyroid isthmus thickness of 5 mm may serve as a clinically relevant threshold, as lesions exceeding this size are associated with a higher risk of extrathyroidal extension. Our previous study demonstrated that recurrence rates were comparable between patients with isthmic PTMCs ≤ 5 mm and > 5 mm who underwent total thyroidectomy [5]. However, it remains unclear whether this threshold is applicable to patients undergoing isthmusectomy. In this study, we compared patients with isthmic PTMC who underwent isthmusectomy based on tumor size (≤ 5 mm vs. > 5 mm) and observed some differences in clinicopathological characteristics and prognostic risk factors, which may provide insights for selecting appropriate candidates for this procedure. The overall recurrence rate in the > 5 mm group was 7.5%, compared with 1.1% in the ≤ 5 mm group (*p* = 0.028).

In the present study, although all patients were classified as cN0 based on preoperative evaluation, postoperative pathological examination confirmed CLNM in 61 patients (30.3%). Previous studies have demonstrated that CLNM is significantly associated with disease recurrence [27–29]. By comparing the clinicopathological variables of patients with and without recurrence, it was observed that patients with recurrence had a significantly higher rate of CLNM (77.8%) and a greater number of metastatic CLNs (*p* < 0.001). These findings indicate that CLNM is a critical prognostic determinant in patients with solitary isthmic PTMC who undergo isthmusectomy. According to the 2025 ATA guidelines, prophylactic CLNs dissection is not routinely recommended for most small, noninvasive, cN0 PTCs and is not a universally accepted standard practice [12]. It is increasingly recognized that lymph nodes identified through routine prophylactic dissection do not necessarily confer a high risk of recurrence unless specific pathological features are present, including the size of metastatic foci, the number of metastatic CLNs, and the presence of extranodal extension [30]. Isthmusectomy combined with bilateral prophylactic CLNs dissection is not a universally recommended procedure fully compliant with ATA guidelines. Prophylactic CLNs dissection was selectively performed at our institution to facilitate accurate nodal staging, considering the high incidence of occult CLN metastasis in isthmic PTMC and the limitations of preoperative imaging.

According to the ATA guidelines, the presence of more than five metastatic CLNs is classified as an intermediate risk for recurrence. Interestingly, the present study shows that the presence of three or more metastatic CLNs was also associated with significantly poorer RFS. ROC analysis showed that the number of metastatic CLNs predicted recurrence in isthmic PTMC, with an AUC of 0.841 and an optimal cut off of > 3 (sensitivity 77.8%, specificity 92.2%). Furthermore, Kaplan–Meier survival analysis also indicated significant differences in RFS between patients with > 3 and ≤ 3 metastatic CLNs (*p* < 0.001). Multivariable analysis indicated that having > 3 metastatic CLNs was an independent risk factor for recurrence (HR = 5.298; 95% CI: 1.473–19.060; *p* < 0.001). These results highlight the critical importance of central compartment lymph node status in determining prognosis. Thus, preoperative assessment of CLNM is essential for guiding individualized primary approaches, including surgical extent and the scope of central neck dissection. Unfortunately, accurate preoperative detection of CLNM is challenging due to the small size of metastatic nodes and their anatomical concealment by adjacent structures. Alabousi et al. reported that the preoperative sensitivity of ultrasonography for detecting CLNM was 28% [31]. Our study found that although the ≤ 5 mm group had a lower incidence of CLNM compared with the > 5 mm group (*p* = 0.009), CLNM was still present in 21.3% of patients in the ≤ 5 mm group. Therefore, bilateral central neck dissection may be considered for patients with isthmic PTMC undergoing isthmusectomy.

The preoperative identification of gETE is a reliable predictor of disease progression and a prognostic factor that remains valid regardless of tumor size. For patients with gETE, total thyroidectomy is generally recommended. Therefore, in this study, patients with isthmic PTMC and gETE identified preoperatively or intraoperatively were excluded. In the 8th edition of the AJCC/TNM cancer staging system, mETE is classified as an intrathyroidal disease and is no longer considered an independent prognostic factor for disease recurrence or mortality [32]. Castagna et al. suggested that, in the absence of other unfavorable features, small tumors with mETE can be classified and managed as low-risk disease [32]. Our results demonstrated that 56.7% of patients with isthmic PTMC had mETE. The incidence of mETE was higher in the > 5 mm group compared with the ≤ 5 mm group (70.1% vs. 41.5%; *p* < 0.001). However, Kaplan–Meier survival analysis indicated that mETE had no significant influence on prognosis (*p* = 0.983). Therefore, for patients with isthmic PTMC undergoing isthmusectomy, the presence of mETE on postoperative pathological examination does not necessitate completion thyroidectomy.

Due to the relatively high rates of mETE (56.7%) and CLNM (30.3%) observed in this study, postoperative TSH suppression therapy was applied in accordance with the 2015 ATA guidelines. Although all patients were categorized as low- to intermediate-risk, these pathological features indicated a non-negligible risk of recurrence. Previous studies have suggested that isthmic papillary thyroid carcinoma may carry a higher recurrence risk than tumors located in the thyroid lobes. Based on these considerations, an initial TSH target of 0.1–0.5 mU/L was applied during the first postoperative year, followed by gradual relaxation to 0.5–2 mU/L according to the individual clinical course and surveillance findings. This may suggest that, for some low-risk patients, TSH suppression could be more intensive than necessary, reflecting a center-specific management strategy rather than a universally applied approach fully consistent with ATA guidelines.

The BRAF^V600E^ mutation occurs frequently in PTC, with a frequency ranging from 25% to 82.3% [33]. Notably, studies have demonstrated that the prevalence of the BRAF^V600E^ mutation is higher in Asian populations than in Western cohorts [34]. Previous studies have suggested that the BRAF^V600E^ mutation is associated with aggressive clinicopathological features and poorer outcomes in PTC [35]. However, recent reports have demonstrated that the BRAF^V600E^ mutation is not associated with adverse outcomes [36, 37]. To date, there have been no studies specifically investigating the BRAF^V600E^ mutation in isthmic PTC. In the present study, the prevalence of the BRAF^V600E^ mutation in isthmic PTMC was 74.6%. Notably, the BRAF^V600E^ mutation rate in the ≤ 5 mm group was significantly lower than that in the > 5 mm group (66.0% vs. 82.2%; *p* = 0.008). Kaplan–Meier survival analysis revealed no significant association between the BRAF^V600E^ mutation and RFS (*p* = 0.069). Multivariable analysis further indicated that the BRAF^V600E^ mutation was not an independent risk factor for recurrence in isthmic PTMC. Therefore, the presence of the BRAF^V600E^ mutation, in the absence of high-risk histological features, does not significantly influence the recurrence of isthmic PTMC.

Drawing on the clinical findings of this study, a preliminary management framework for isthmic PTMC can be proposed to support individualized surgical planning. Preoperatively, isthmusectomy may be appropriate for solitary lesions ≤ 5 mm, while tumors larger than 5 mm warrant careful evaluation and thorough patient counseling regarding potential recurrence risk. After surgery, postoperative histopathologic assessment can help refine risk stratification. In particular, the presence of more than three metastatic CLNs may indicate the need for closer clinical and ultrasonographic follow-up. In contrast, mETE or the BRAF^V600E^ mutation, in the absence of other high-risk features, may not necessarily require completion thyroidectomy and could allow for a more conservative follow-up strategy. Overall, this stratified approach may assist clinicians in tailoring the extent of surgery and follow-up intensity according to individual patient risk, though these observations are preliminary and should be validated in future prospective studies.

The present study has several limitations. First, the sample size of patients with isthmic PTMC was relatively small, which may have introduced selection bias. Second, although the study spanned six years, the median follow-up duration was 30.0 months; this may be insufficient to capture late recurrences. Future multicenter, prospective studies with larger sample sizes and longer follow-up durations are needed to validate these findings.

## Conclusion

In conclusion, our findings suggest that tumor size > 5 mm and the presence of CLNM, particularly when > 3 nodes are involved, may be associated with a higher risk of recurrence after isthmusectomy in isthmic PTMC. In contrast, mETE and BRAF^V600E^ mutation appeared to have limited impact on prognosis. These observations could help guide surgical decision-making through individualized approaches to the extent of resection based on careful assessment of tumor characteristics, although further prospective validation is warranted.

## Data Availability

The datasets generated and/or analyzed during the current study are not publicly available because First Affiliated Hospital, College of Medicine, Zhejiang University owns the data. However, the datasets are available from the corresponding author on reasonable request.

## Abbreviations

PTC: Papillary thyroid carcinoma
PTMC: Papillary thyroid microcarcinoma
cN0: Clinically node-negative
ROC: Receiver operating characteristic
ETE: Extrathyroidal extension
Mete: Microscopic extrathyroidal extension
gETE: Gross extrathyroidal extension
CLNM: Central lymph node metastasis
CLN: Central lymph node
RFS: Recurrence-free survival
ATA: American Thyroid Association
FNAB: Fine-needle aspiration biopsy
TSH: Thyroid stimulating hormone

## References

[1] Baloch ZW, et al. Overview of the 2022 Who Classification of Thyroid Neoplasms. Endocr Pathol. 2022;33:27–63. 35288841 10.1007/s12022-022-09707-3

[2] Hahn SY, Han BK, Ko EY, Shin JH, Ko ES. Ultrasound Findings of Papillary Thyroid Carcinoma Originating in the Isthmus: Comparison with Lobe-Originating Papillary Thyroid Carcinoma. AJR Am J Roentgenol. 2014;203:637–42. 25148169 10.2214/AJR.13.10746

[3] Lim ST, Jeon YW, Suh YJ. Correlation between Surgical Extent and Prognosis in Node-Negative, Early-Stage Papillary Thyroid Carcinoma Originating in the Isthmus. World J Surg. 2016;40:344–9. 26446448 10.1007/s00268-015-3259-2

[4] Zhu F, Li F, Xie X, Wu Y, Wang W. Investigating the Impact of Tumor Location and Size on the Risk of Recurrence for Papillary Thyroid Carcinoma in the Isthmus. Cancer Med. 2023;12:13290–99. 37132252 10.1002/cam4.6023 PMC10315713

[5] Zhu F, et al. Differences in the Clinical Characteristics of Papillary Thyroid Microcarcinoma Located in the Isthmus =5 Mm and 5 mm in Diameter. Front Oncol. 2022;12:923266. 35978829 10.3389/fonc.2022.923266 PMC9376609

[6] Lee YS, et al. Papillary Carcinoma Located in the Thyroid Isthmus. World J Surg. 2010;34:36–9. 20020291 10.1007/s00268-009-0298-6

[7] Lei J, Zhu J, Li Z, Gong R, Wei T. Surgical Procedures for Papillary Thyroid Carcinoma Located in the Thyroid Isthmus: An Intention-to-Treat Analysis. Onco Targets Ther. 2016;9:5209–16. 27578987 10.2147/OTT.S106837 PMC5001660

[8] Chang YW, et al. Extent of Central Lymph Node Dissection for Papillary Thyroid Carcinoma in the Isthmus. Ann Surg Treat Res. 2018;94:229–34. 29732353 10.4174/astr.2018.94.5.229 PMC5931932

[9] Shuai Y, et al. Surgical Extent of Central Lymph Node Dissection for Papillary Thyroid Carcinoma Located in the Isthmus: A Propensity Scoring Matched Study. Front Endocrinol (Lausanne). 2021;12:620147. 34211434 10.3389/fendo.2021.620147 PMC8240638

[10] Gui Z, et al. Comparison of Outcomes Following Thyroid Isthmusectomy, Unilateral Thyroid Lobectomy, and Total Thyroidectomy in Patients with Papillary Thyroid Microcarcinoma of the Thyroid Isthmus: A Retrospective Study at a Single Center. Med Sci Monit. 2020;26:e927407. 33351790 10.12659/MSM.927407 PMC7763914

[11] Kwon O, Lee S, Bae JS, Jung CK. Thyroid Isthmusectomy with Prophylactic Central Compartment Neck Dissection Is a Feasible Approach for Papillary Thyroid Cancer on the Isthmus. Ann Surg Oncol. 2021;28:6603–12. 33768393 10.1245/s10434-021-09833-y

[12] Ringel MD, et al. 2025 American Thyroid Association Management Guidelines for Adult Patients with Differentiated Thyroid Cancer. Thyroid. 2025;35:841–985. 40844370 10.1177/10507256251363120 PMC13090833

[13] Won HS, et al. Location and Morphometry of the Thyroid Isthmus in Adult Korean Cadavers. Anat Sci Int. 2013;88:212–6. 23818140 10.1007/s12565-013-0187-9

[14] Grogan RH, et al. A Study of Recurrence and Death from Papillary Thyroid Cancer with 27 Years of Median Follow-Up. Surgery. 2013;154:1436–46. discussion 46 – 7. 24075674 10.1016/j.surg.2013.07.008

[15] Untch BR, et al. Oncologic Outcomes after Completion Thyroidectomy for Patients with Well-Differentiated Thyroid Carcinoma. Ann Surg Oncol. 2014;21:1374–8. 24366419 10.1245/s10434-013-3428-1

[16] Tran TV, et al. All-Cause and Cause-Specific Mortality among Low-Risk Differentiated Thyroid Cancer Survivors in the United States. Thyroid. 2024;34:215–24. 38149602 10.1089/thy.2023.0449 PMC10884550

[17] Lee YC, Na SY, Chung H, Kim SI, Eun YG. Clinicopathologic Characteristics and Pattern of Central Lymph Node Metastasis in Papillary Thyroid Cancer Located in the Isthmus. Laryngoscope. 2016;126:2419–21. 27098428 10.1002/lary.25926

[18] Goldfarb M, Rodgers SS, Lew JI. Appropriate Surgical Procedure for Dominant Thyroid Nodules of the Isthmus 1 Cm or Larger. Arch Surg. 2012;147:881–4. 22987188 10.1001/archsurg.2012.728

[19] Karatzas T, Charitoudis G, Vasileiadis D, Kapetanakis S, Vasileiadis I. Surgical Treatment for Dominant Malignant Nodules of the Isthmus of the Thyroid Gland: A Case Control Study. Int J Surg. 2015;18:64–8. 25900600 10.1016/j.ijsu.2015.04.039

[20] Perros P, et al. Guidelines for the Management of Thyroid Cancer. Clin Endocrinol (Oxf). 2014;81(Suppl 1):1–122. 24989897 10.1111/cen.12515

[21] Yuan Q, et al. Prognosis and Postoperative Complications of Wide-Field Isthmusectomy for Node-Negative Papillary Thyroid Carcinoma Limited to the Isthmus. J Otolaryngol Head Neck Surg. 2025;54:19160216251348423. 40560773 10.1177/19160216251348423 PMC12198579

[22] Park H, Harries V, McGill MR, Ganly I, Shah JP. Isthmusectomy in Selected Patients with Well-Differentiated Thyroid Carcinoma. Head Neck. 2020;42:43–9. 31589005 10.1002/hed.25968 PMC7485011

[23] Hartl DM et al. Thyroid Lobectomy for Low to Intermediate Risk Differentiated Thyroid Cancer. Cancers (Basel). 2020;12. 10.3390/cancers12113282 PMC7694652 33171949

[24] Rossi L, Paternoster M, Cammarata M, Bakkar S, Miccoli P. Levothyroxine Therapy in Thyroidectomized Patients: Ongoing Challenges and Controversies. Front Endocrinol (Lausanne). 2025;16:1582734. 40491597 10.3389/fendo.2025.1582734 PMC12146200

[25] Yaniv D, et al. Quality of Life Following Lobectomy Versus Total Thyroidectomy Is Significantly Related to Hypothyroidism. J Surg Oncol. 2022;126:640–48. 35689620 10.1002/jso.26983 PMC9544480

[26] Bakkar S, et al. Postoperatively Determined High-Risk Histopathologic Features in Papillary Thyroid Carcinoma Initially Eligible for Thyroid Lobectomy: A Game Changer. Endocrine. 2021;74:611–15. 34110601 10.1007/s12020-021-02788-w

[27] Ma B, Wang Y, Yang S, Ji Q. Predictive Factors for Central Lymph Node Metastasis in Patients with Cn0 Papillary Thyroid Carcinoma: A Systematic Review and Meta-Analysis. Int J Surg. 2016;28:153–61. 26944586 10.1016/j.ijsu.2016.02.093

[28] Bernet V. Approach to the Patient with Incidental Papillary Microcarcinoma. J Clin Endocrinol Metab. 2010;95:3586–92. 20685885 10.1210/jc.2010-0698

[29] Wang L, et al. Predicting Central Cervical Lymph Node Metastasis in Papillary Thyroid Carcinoma with Hashimoto’s Thyroiditis: A Practical Nomogram Based on Retrospective Study. PeerJ. 2024;12:e17108. 38650652 10.7717/peerj.17108 PMC11034492

[30] Bakkar S, et al. Verifying the Oncologic Rationale of Prophylactic Central Compartment Neck Dissection in the Management of Papillary Thyroid Carcinoma Using a Pathologic Spectrum of Nodal Metastases Characteristics. A Prospective Comparative Study. Endocrine. 2025;89:119–24. 40024949 10.1007/s12020-025-04209-8

[31] Alabousi M, et al. Diagnostic Test Accuracy of Ultrasonography Vs Computed Tomography for Papillary Thyroid Cancer Cervical Lymph Node Metastasis: A Systematic Review and Meta-Analysis. JAMA Otolaryngol Head Neck Surg. 2022;148:107–18. 34817554 10.1001/jamaoto.2021.3387 PMC8613701

[32] Parvathareddy SK, et al. Microscopic Extrathyroidal Extension Results in Increased Rate of Tumor Recurrence and Is an Independent Predictor of Patient’s Outcome in Middle Eastern Papillary Thyroid Carcinoma. Front Oncol. 2021;11:724432. 34926245 10.3389/fonc.2021.724432 PMC8671701

[33] Brumfield A, et al. Prevalence and Clinical Impact of Braf P.V600e Mutation in Papillary Thyroid Carcinoma. Endocr Pathol. 2025;36:13. 40237893 10.1007/s12022-025-09859-y PMC12003545

[34] Rashid FA, Munkhdelger J, Fukuoka J, Bychkov A. Prevalence of Braf(V600e) Mutation in Asian Series of Papillary Thyroid Carcinoma-a Contemporary Systematic Review. Gland Surg. 2020;9:1878–900. 33224863 10.21037/gs-20-430 PMC7667088

[35] Xing M, et al. Association between Braf V600e Mutation and Recurrence of Papillary Thyroid Cancer. J Clin Oncol. 2015;33:42–50. 25332244 10.1200/JCO.2014.56.8253 PMC4268252

[36] Lai HF, et al. Braf V600e Mutation Lacks Association with Poorer Clinical Prognosis in Papillary Thyroid Carcinoma. Ann Surg Oncol. 2024;31:3495–501. 38300401 10.1245/s10434-024-14935-4

[37] Wei X, et al. Risk and Prognostic Factors for Braf(V600e) Mutations in Papillary Thyroid Carcinoma. Biomed Res Int. 2022;2022:9959649. 35647194 10.1155/2022/9959649 PMC9132653

